# Supplementary material for: Short-Term Autophagy Preconditioning Upregulates the Expression of COX2 and PGE2 and Alters the Immune Phenotype of Human Adipose-Derived Stem Cells In Vitro
Source: Cells. 2022 Apr 19;11(9):1376. doi: 10.3390/cells11091376 (PMC9101706; doi:10.3390/cells11091376)
Supplement: Supplementary file 1 [file cells-11-01376-s001.zip › Supplementary Figure S1.pdf]

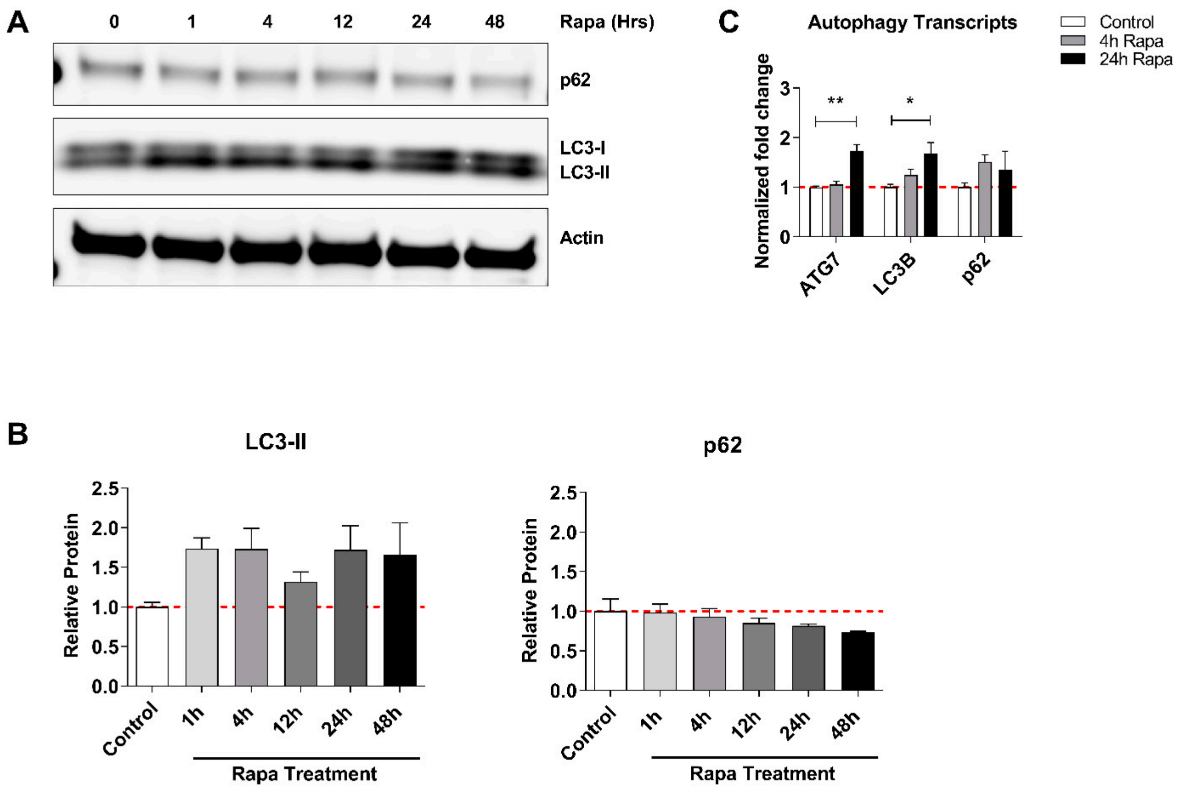

Supplementary Figure S1: *Rapamycin induces autophagy in hASCs.*

(**A**) Representative Western Blot (WB) of p62, LC3-II, and β-Actin in Rapa-ASCs. (**B**) Densitometric analysis of WB. Data are presented as means ± SEM of 3 independent experiments. (**C**) Transcriptional analysis of Atg7, LC3B, and p62 in Rapa-ASCs. Data are presented as means ± SEM of 4 independent experiments. Statistical analysis was performed using one-way analysis of variance (ANOVA) and Tukey's post-hoc multiple comparisons. Statistical differences between the means are marked with *$p < 0.05$, **$p < 0.01$, ***$p < 0.001$. Abbreviations: Rapa, Rapamycin; p62, ubiquitin-binding protein p62; LC3B, microtubule-associated proteins 1A/1B light chain 3B; LC3-II, LC3-phosphatidylethanolamine conjugate; ATG7, Autophagy Related 7.
